# CTX-M–producing Non-Typhi *Salmonella* spp. Isolated from Humans, United States

**DOI:** 10.3201/eid1701.100511

**Published:** 2011-01

**Authors:** Maria Sjölund-Karlsson, Rebecca Howie, Amy Krueger, Regan Rickert, Gary Pecic, Kathryn Lupoli, Jason P. Folster, Jean M. Whichard

**Affiliations:** Author affiliations: Centers for Disease Control and Prevention, Atlanta, Georgia, USA (M. Sjölund-Karlsson, J.M. Whichard);; Atlanta Research and Education Foundation, Decatur, Georgia, USA (R. Howie, A. Krueger, R. Rickert, G. Pecic, K. Lupoli, J.P. Folster)

**Keywords:** Salmonella, antimicrobial resistance, CTX-M beta-lactamase, bacteria, United States, dispatch

## Abstract

CTX-M–type β-lactamases are increasing among US *Enterobacteriaceae* isolates. Of 2,165 non-Typhi *Salmonella* isolates submitted in 2007 to the National Antimicrobial Resistance Monitoring System, 100 (4.6%) displayed elevated MICs (>2 mg/L) of ceftriaxone or ceftiofur. Three isolates (serotypes Typhimurium, Concord, and I 4,5,12:i:–) contained *bla*_CTX-M-5_, *bla*_CTX-M-15_, and *bla*_CTX-M-55/57_, respectively.

Severe non-Typhi *Salmonella* (NTS) infections are commonly treated with fluoroquinolones such as ciprofloxacin or, in children, with extended-spectrum cephalosporins such as ceftriaxone. The emergence of *Salmonella* spp. isolates that display resistance to extended-spectrum cephalosporins is of increasing public health concern. In the United States, almost all resistance to extended-spectrum cephalosporins among *Salmonella* spp. isolates is caused by AmpC-type β-lactamases; extended-spectrum β-lactamases (ESBLs), including cefotaximases (CTX-M), rarely have been reported. Likewise, among other *Enterobacteriaceae* isolates in the United States, CTX-M enzymes have been considered rare until recently.

In 2007, Lewis et al. reported that CTX-M was the predominant ESBL among *Enterobacteriaceae* isolates in a US health care system (San Antonio, TX) (*1*). Among the ESBL-producing isolates collected, CTX-M enzymes increased in prevalence from 25% in 2002 to 70% in 2006. The emergence was observed mainly in urinary tract isolates of *Escherichia coli*, and the predominating enzyme was CTX-M-15 (*1*). Similarly, a US study investigating clinical samples of *Enterobacteriaceae* submitted to The Hospital of the University of Pennsylvania (Philadelphia, PA) in 2007 reported that 48% of cephalosporin-resistant *E. coli* isolates were CTX-M positive (*2*). Furthermore, CTX-M enzymes, and CTX-M-15 in particular, were common among ESBL-producing *Enterobacteriaceae* isolates collected at 15 US medical centers participating in the Meropenem Yearly Susceptibility Test Information Collection Program during 2007 (*3*).

In the United States, the National Antimicrobial Resistance Monitoring System (NARMS) has systematically monitored antimicrobial susceptibility of NTS since 1996. This program is a collaborative effort between the Centers for Disease Control and Prevention (CDC), the Food and Drug Administration, and the US Department of Agriculture. Reports on increased prevalence of CTX-M enzymes among *Enterobacteriaceae* isolates in the United States prompted us to investigate CTX-M enzymes among NARMS NTS isolates collected from humans in 2007.

## The Study

In 2007, public health laboratories in all US state health departments submitted every twentieth NTS isolated from humans to CDC for susceptibility testing by NARMS. MICs were determined by using broth microdilution (Sensititer, Trek Diagnostics, Westlake, OH, USA) and interpreted according to Clinical Laboratory Standards Institute criteria, where available.

Of the 2,165 human NTS isolates submitted to NARMS in 2007, a total of 100 (4.6%) displayed elevated MICs (>2 mg/L) of ceftriaxone or ceftiofur, extended-spectrum cephalosporins used in human and veterinary medicine, respectively. Genomic DNA prepared from the 100 isolates and a PCR screen obtained by using degenerate primers capable of detecting all CTX-M enzymes identified 3 positive isolates, including serotypes Typhimurium; I 4,5,12:i:–; and Concord (*4*). Most (66%) of the remaining 97 isolates harbored a *bla*_CMY_ gene.

The 3 CTX-M–producing *Salmonella* spp. infections occurred in 2 female patients (8 months of age and 72 years of age) and 1 male patient (1 year of age). Interviews were available for 2 patients, the 8-month-old (her parents) and the 72-year-old; in both instances, gastrointestinal symptoms with diarrhea were reported, and medical care was sought. Both patients received antimicrobial drug treatment (azithromycin and levofloxacin, respectively). The 72-year-old patient had traveled internationally before illness onset; the 8-month-old patient, who was infected with *S.* *enterica* serovar Concord, was an adoptee from Ethiopia.

All 3 isolates displayed resistance to β-lactams and extended-spectrum cephalosporins (Table). The *S.* *enterica* serovar Typhimurium and Concord isolates displayed additional multiresistance phenotypes. In addition, the serovar Typhimurium isolate displayed resistance to the quinolone nalidixic acid, a resistance phenotype associated with decreased susceptibility to fluoroquinolones. The serovar Typhimurium and Concord isolates showed decreased susceptibility to ciprofloxacin (MIC 0.25 mg/L and 0.125 mg/L, respectively). PCR for the plasmid-mediated mechanisms *qnrA,B,S* and *aac(6′)Ib-cr* showed a *qnrA* gene in the serovar Concord isolate. Sequencing confirmed *qnrA1*.

**Table:** Characteristics of non-Typhi Salmonella isolates harboring blaCTX-M genes reported to the National Antimicrobial Resistance Monitoring System, United States, 2007*

Isolate no.	Serotype	Submitting state	Resistance pattern	CTX MIC, mg/L	bla genes	qnr gene	Foreign travel association	Transferable
AM33608	Typhimurium	SC	AMP, AUG, CHL, CTX, NAL, SUL, SXT, XNL	64	CTX-M-5, OXA-1	–	ND	No
AM32667	I 4,5,12:i:–	CT	AMP, CTX, XNL	64	CTX-M-55/57	–	No	Yes
AM32764	Concord	OR	AMP, CHL, CTX, GEN, STR, SUL, SXT, TET, XNL	64	CTX-M-15, SHV-12	qnrA1	Ethiopia	No

*CTX, ceftriaxone; AMP, ampicillin; AUG, amoxicillin-clavulanic acid; CHL, chloramphenicol; NAL, nalidixic acid; SUL, sulfamethoxazole or sulfisoxazole; SXT, trimethoprim–sulfamethoxazole; XNL, ceftiofur; –, none detected; ND, not determined; GEN, gentamicin; STR, streptomycin; TET, tetracycline.

Group-specific PCR primers were used to characterize the presumed *bla*_CTX-M_ genes (*5*). *S.* *enterica* serovar Concord and I 4,5,12:i:– harbored group I enzymes, whereas the *S.* *enterica* serovar Typhimurium isolate harbored a group II enzyme. Sequencing showed *bla*_CTX-M-15_ in the serovar Concord isolate, *bla*_CTX-M-5_ in the serovar Typhimurium isolate, and *bla*_CTX-M-55/57_ in the serovar I 4,5,12:i:– isolate. Presence of other β-lactamase–encoding genes (*bla*_TEM_, *bla*_SHV_, *bla*_CMY_, *bla*_PSE_, and *bla*_OXA_) was investigated by using PCR (*6**–**9*). Amplification and sequencing confirmed a *bla*_OXA-1_ and a *bla*_SHV-12_ gene in the serovar Typhimurium and Concord isolates, respectively (*7**,**8*).

The genetic environment of each *bla*_CTX-M_ gene was investigated by using PCR aimed at identifying insertion elements IS*EcpI*, IS*26*, and CR1 (formerly known as orf513) (*10*). Amplification and sequencing of the PCR products confirmed the IS*Ecp1* element upstream of each *bla*_CTX-M_ gene (*10*). In addition to IS*Ecp1*, an IS*26* element was detected upstream of the *bla*_CTX-M-5_ and *bla*_CTX-M-15_ genes.

To determine whether the CTX-M enzymes were plasmid borne, we extracted and transformed plasmids into electrocompetent *E. coli* DH10B. The *bla*_CTX-M-55/57_ gene transferred to *E. coli*; repeated attempts to transfer the *bla*_CTX-M-5_ and *bla*_CTX-M-15_ genes were unsuccessful. PCR amplification and plasmid pulsed-field gel electrophoresis confirmed the presence of the *bla*_CTX-M-55/57_ gene on a 70-kb plasmid in the transformant. The plasmid was not typeable by PCR-based incompatibility/replicon typing.

## Conclusions

We describe 3 CTX-M–producing isolates of NTS collected from humans in the United States during 2007. CTX-M–producing *Salmonella* spp. previously have been reported among the NARMS collection of human isolates. The first isolate was *S.* *enterica* serovar Typhimurium from a 3-month-old child in Georgia in 2003 (*11*). This infection was considered domestically acquired because the child’s family did not report a history of international travel. In addition, a CTX-M–15–producing isolate of *S.* *enterica* serovar Concord was identified among NARMS NTS collected in 2006 (*12*). However, in contrast to the previous case, this infection most likely was acquired abroad because the patient reported travel to Ethiopia in conjunction with illness onset.

At least 1 of the infections described in the present study probably was acquired abroad; the CTX-M-15–producing *S.* *enterica* serovar Concord isolate was isolated from an adopted child from Ethiopia. Thus, both instances of CTX-M–producing Concord isolates identified in NARMS thus far have been associated with travel to Ethiopia. The emergence of CTX-M-15–producing serovar Concord infections among Ethiopian adoptees has been described previously (*13*). In addition, the emergence of serovar Concord isolates that produced CTX-M-15, SHV-12, and QnrA1 was recently described (*14*).

The fact that *bla*_CTX-M_ genes commonly are located on plasmids and in conjunction with mobile genetic elements such as IS*EcpI* most likely has contributed to their dissemination. The *bla*_CTX-M-55/57_ gene that was transferable to *E. coli* in the present study was located on a 70-kb plasmid. The fact that the *bla*_CTX-M-5_ and *bla*_CTX-M-15_ gene did not transfer might suggest chromosomal locations. In fact, Fabre et al. found that most CTX-M-15–producing *S.* *enterica* serovar Concord isolates studied harbored the *bla*_CTX-M_ gene on the chromosome (*13*).

The recently reported increase in CTX-M–producing *Enterobacteriaceae* in the United States raises concern. First, a reservoir of ESBLs and CTX-M genes among *E. coli* and *Klebsiella* spp. constitutes a risk factor for increased spread of resistance to other pathogenic bacteria, including *Salmonella* spp. Second, use of cephalosporins to treat serious ESBL-producing bacterial infections has been associated with high rates of treatment failure (*15*). Thus, an increase in CTX-M–producing *Salmonella* spp. strains is likely to directly affect treatment, especially among children for whom use of fluoroquinolones is contraindicated. Moreover, the decreased susceptibility to ciprofloxacin of the serovar Typhimurium and Concord isolates in the present study raises concern about the emergence of isolates showing concurrent resistance to both extended-spectrum cephalosporins and fluoroquinolones. Continued surveillance of resistant bacteria, in combination with prudent use of antimicrobial agents in animals and humans, is crucial for limiting further spread of CTX-M–producing isolates of *Enterobacteriaceae* in the United States.
